# Emotional regulation and attachment in adolescents with anorexia nervosa

**DOI:** 10.1192/j.eurpsy.2021.945

**Published:** 2021-08-13

**Authors:** O. Khaustova, L. Sak

**Affiliations:** Medical Psychology, Psychosomatic Medicine & Psychotherapy, Bogomolets National Medical University, Kiyv, Ukraine

**Keywords:** anorexia nervosa, Emotional Regulation, attachment, DERS

## Abstract

**Introduction:**

The aim of the present study was to investigate emotional regulation and attachment in adolescents with anorexia nervosa (AN).

**Objectives:**

Anorexia Nervosa (AN) is an eating disorder (ED) characterized by self-starvation driving by weight, shape, and eating concerns and extreme dread of food, eating, and normal body weight. Dysfunctional emotional processing and regulation play an important role in the development and maintenance of eating disorders (EDs). Difficulties with emotional awareness and regulation in EDs are hypothesized to have their origins in childhood attachment.

**Methods:**

The study population (N=20) consists of two research groups of patients with AN (group A) and general population controls (group B), matched for gender and age. The age of patients was 12-18 years. All adolescents were female. This study examined the attachment states of mind, assessed by the Adult Attachment Interview (AAI), and emotion regulation difficulties, measured by the Difficulties in Emotion Regulation Scale (DERS).

**Results:**

Group A reported significantly higher attachment insecurity (82% vs 50%) than group B. Group A show higher DERS total (nonacceptance, goals, and impulsivity scores) than group B.

**Conclusions:**

Study results show a crucial role of attachment insecurity and emotional dysregulation in the development and maintenance of AN. Developing interventions to improve emotional management skills in the treatment of patients with AN can be an important component in improving treatment outcomes.

